# Knox and Mitchell's Engravings of the Bones, Arteries, and Nerves

**Published:** 1831-01-01

**Authors:** 


ENGRAVINGS OF THE BONES, ARTERIES, AND NERVES, COPIED FROM THE WORKS OF SCARPA, SOEMMERING, &c. By EDWARD MITCHELL, Engraver. With an Explanatory Letter-press by Dr. KNOX.

In a former number of our Periscope, (July 1829,) we alluded briefly to a series of plates in progress of publication, intended to illustrate the anatomy of the human body. These prints, we are happy to learn, have met with the encouragement they deserve, and Mr. Mitchell and Dr. Knox the ingenious engraver and editor, may congratulate themselves on having accomplished that object, which was so ardently desired, both by practitioners and students.

We have already stated, that these illustrations are taken, for the most part, from the splendid engravings of Haller, Camper, Scarpa, &c. and will, when completed, form a kind of miniature edition of Caldani, with this advantage however, that all the anatomical discoveries made since his time will be embodied. The price of the work is extremely moderate, too moderate indeed, in our opinion, to afford any remuneration to the conductors of it.

Before alluding specifically to any of the plates, we would remark that Dr. Knox, with great good sense, has avoided the common error of book-makers, and has not attempted to alter or amend any of the splendid designs of Anderloni or Camper, nor has he clogged the descriptive part of the work with mawkish quotations and incorrect original physiological and pathological observations. A simple and perspicuous account of the parts is all that the surgeon or student wants in a publication of this kind, and we can conscientiously recommend the engravings of Mr. Mitchell as safe and sure guides to the knife of the anatomist and surgeon, which is more than we can do of some other works on the same subject, which must have cost their compilers a pretty penny in the way of newspaper-puffs and advertisements.

The work has very properly been divided into three parts, each of which may be had separately.

It is principally from the great work of Sue, and from bones in the collection of the late Dr. Barclay, that the osteological engravings have been made; the delineations are extremely accurate, and convey just notions of the situation of even the most minute foramina and processes, but as the skeleton itself is, or should be, in the possession of every medical student, we shall not dwell longer on this department.

To Plates V. VI. VII. and X. of the arteries, we would direct the especial attention of the student, as they display in a distinct and simple chain, not merely the arterial, but also the myological anatomy of the head, neck, and throat, in different points of view. These four plates should be in the hands of every student who frequents the dissecting room. They will save him much labour and anxiety in the examination of these most important regions, and will enable him to comprehend, more easily than he otherwise would do, the descriptions given in the different vade mecums and elementary treatises on anatomy.

In our own time, we were wont to dissect with the aid of Haller's great work on the arteries, and Cloquet's no less ponderous volume, but we did so in fear and trembling, for it was a difficult matter to preserve them from the rude grasp of students, more enthusiastic perhaps than ourselves, and who were not aware of their intrinsic value.

The arterial anatomy of the hand and arm occupies another series of plates, which will be found by the young student to be of much value when he is dissecting the body for the first time. The course and ramifications of the brachial artery are distinctly marked, and their connexion with the muscles and tendons accurately displayed.

In the third division of the work is contained all that is known relative to the distribution of the nerves. The plates are most beautifully executed, and are faithfully reduced from the splendid engravings of Anderloni and other eminent artists.

With this book, and one of the many excellent anatomical manuals in his hands, the student will find himself *almost* independent of a demonstrator, at least in so far as mere *descriptive* anatomy is concerned. By studying it with care before going to—when in—and after leaving the dissecting room, he will soon feel sufficient confidence to dissect with the aid of the manual alone, and by having studied the parts in connection with the plates, he cannot fail to acquire a kind of mnemonical memory, which is of itself a matter of no small importance to the practical surgeon.

